# The Effect of Biologic Agents on Steatotic Liver Disease in Patients with Inflammatory Bowel Disease: A Prospective, Open-Label Comparative Trial

**DOI:** 10.3390/ph17111432

**Published:** 2024-10-25

**Authors:** Apostolis Papaefthymiou, Styliani Sarrou, Konstantinos Pateras, Ilias D. Vachliotis, Georgios Agrotis, Ioanna-Konstantina Sgantzou, Georgios Perifanos, Andreas Kapsoritakis, Matthaios Speletas, Marianna Vlychou, George N. Dalekos, Spyros Potamianos, Antonis Goulas, Jannis Kountouras, Stergios A. Polyzos

**Affiliations:** 1 First Laboratory of Pharmacology, School of Medicine, Aristotle University of Thessaloniki, 54124 Thessaloniki, Greece; ilvachliotis@gmail.com (I.D.V.); agoulas@auth.gr (A.G.); spolyzos@auth.gr (S.A.P.); 2 Department of Gastroenterology, University Hospital of Larissa, School of Medicine, University of Thessaly, 41100 Larissa, Greece; kapsoritakis@uth.gr (A.K.); spotam@med.uth.gr (S.P.); 3 Department of Immunology & Histocompatibility, Faculty of Medicine, University of Thessaly, 41100 Larissa, Greece; ssarrou@uth.gr (S.S.); maspel@med.uth.gr (M.S.); 4 Gastroenterology Private Practice, 41222 Larissa, Greece; kostas_pks@hotmail.com; 5 Department of Gastroenterology, 424 General Military Training Hospital, 56429 Thessaloniki, Greece; 6 Department of Radiology, University General Hospital of Larissa, 41100 Larissa, Greece; g.agrotis@hotmail.com (G.A.); iksgantzou@gmail.com (I.-K.S.); mvlychou@med.uth.gr (M.V.); 7 Research Laboratory of Internal Medicine, Department of Medicine, National Expertise Center of Greece in Autoimmune Liver Diseases, General University Hospital of Larissa, 41110 Larissa, Greece; geoproud1@gmail.com (G.P.); georgedalekos@gmail.com (G.N.D.); 8 Second Medical Clinic, School of Medicine, Ippokration Hospital, Aristotle University of Thessaloniki, 54642 Thessaloniki, Greece; jannis@auth.gr

**Keywords:** biologics, inflammatory bowel diseases, metabolic-dysfunction associated steatotic liver disease, metabolic-dysfunction associated steatohepatitis, nonalcoholic fatty liver disease, nonalcoholic steatohepatitis

## Abstract

Background: Biologic agents used in patients with inflammatory bowel diseases (IBD) may influence the pathophysiology of coexistent metabolic-dysfunction associated steatotic liver disease (MASLD). This study primarily aimed to evaluate the six-month effect of infliximab or vedolizumab vs. no biologics on presumed hepatic steatosis in patients with IBD. Secondary endpoints were their effect on hepatic fibrosis and parameters related to hepatic metabolism. Methods: This prospective, non-randomized, controlled trial assigned adult bio-naïve patients with IBD into three groups: infliximab, vedolizumab, or controls (receiving no biologic). The baseline was the time of the initiation of biologic agents and the endpoint six months later. Hepatic steatosis was evaluated with transabdominal ultrasonography (Hamaguchi score), whereas controlled attenuation parameter (CAP), fatty liver index (FLI), and hepatic steatosis index (HSI) were used as surrogates. Hepatic fibrosis was evaluated with liver stiffness (LS), fibrosis-4 index (FIB-4), and nonalcoholic fatty liver disease (NAFLD) fibrosis score. Results: Sixty-six patients were assigned to infliximab (n = 26), vedolizumab (n = 14), or control (n = 26); At the endpoint, the Hamaguchi score, CAP, FLI, and HSI were not different between groups. LS was not different between groups; however, FIB-4 was increased within all groups, and NAFLD fibrosis score was increased within infliximab and control groups, without significant biologic × time interactions. Conclusions: No positive or adverse effect of infliximab or vedolizumab vs. no biologic agents was shown on presumed hepatic steatosis in patients with IBD, who have not been previously exposed to biologic agents. Although no effect of both biologic agent on LS, a slight but significant increase in FIB-4 and NAFLD fibrosis score warrants further studying.

## 1. Introduction

Inflammatory bowel diseases (IBD) comprise chronic gastrointestinal disorders with systemic manifestations, including metabolic-dysfunction associated steatotic liver disease (MASLD) [1,2,3]. MASLD seems to be common among patients with IBD, potentially increasing the risk of cardiovascular diseases and chronic kidney disease, as well as their overall morbidity [4]. However, the frequency of MASLD among people Crohn’s disease (CD) or ulcerative colitis (UC) varies significantly across studies, reported to be between 1.5% and 55% [2,5,6], with higher rates observed when more accurate diagnostic methods like controlled attenuation parameter (CAP) elastography or magnetic resonance imaging-proton density fat fraction (MRI-PDFF) are used [7].

Experimental and observational studies have identified a range of potential risk factors of MASLD in patients with IBD, categorized into IBD-specific and non-specific factors [7]. Briefly, MASLD is especially common in underweight IBD patients, suggesting that inflammation and impaired gut permeability significantly contribute to the progression of liver disease through the gut–liver axis [8,9,10]. Systemic inflammation also plays a central role in MASLD development, with cytokines, like tumor necrosis factor (TNF), shown to impair insulin signaling and to promote hepatic inflammation and fibrosis through the activation of hepatic stellate cells [8,11,12]. Furthermore, gut microbiota dysbiosis, common in IBD, disrupts the intestinal barrier, allowing harmful bacteria and their products to enter into the liver, triggering immune responses that may contribute to MASLD pathogenesis [13,14,15]. Additionally, aging, obesity, sarcopenia, or their coexistence, known as sarcopenic obesity, which are commonly observed in individuals with IBD, may further increase the risk of MASLD in this population [16,17,18,19,20]. These findings underline the complex interplay between IBD, gut microbiota, and MASLD, and create the need to investigate the clinical translation and implication of their cross-talk. In light of the specific pathogenic factors present in IBD patients that may lead to MAFLD development, we proposed the term IBD metabolic dysfunction-associated fatty liver disease (IBDAFLD) in order to describe a subset of patients who have both MAFLD and IBD [7].

The introduction of biologic agents represents a milestone in the management of patients with IBD; we previously speculated that biologic agents may also impact IBDAFLD, since the pathophysiologic mechanisms of IBD and IBDAFLD may cross-talk [7]. Anti-TNF agents, including infliximab, which are particularly used in moderate-to-severe IBD, are hypothesized to alleviate inflammation and improve hepatic steatosis and inflammation. This may occur by reducing the reliance on glucocorticosteroids and modulating inflammatory pathways linked to the pathogenesis of MASLD [12,21,22]. Clinical data have reinforced this concept, with patients with IBD on anti-TNF therapy exhibiting reduced hepatic steatosis markers compared to those not on biologics, suggesting that TNF inhibition might mitigate hepatic fat accumulation [23]. This effect may be attributed to the anti-inflammatory action of TNF blockers, which reduces hepatic inflammation and steatosis in MASLD patients [24]. However, the few existing clinical studies have produced controversial results; some of them reported neutral or marginally beneficial impact of anti-TNF agents on MASLD, while others reported higher prevalence of MASLD after treatment, potentially associated with weight gain and an increase in visceral adiposity after achieving remission of IBD [25,26,27]. Vedolizumab, an agent targeting the α4β7 integrin, was also initially believed to influence hepatic metabolism, through the α4β7 integrin and its ligand, mucosal addressin cell adhesion molecule (MAdCAM)-1, but limited clinical evidence also remains controversial with some retrospective studies showing an inverse association of vedolizumab with hepatic steatosis [6,20,28]. The role of α4β7 integrin in hepatic fibrosis and MASLD may be more complex than initially thought. It has been hypothesized that blocking α4β7/MAdCAM-1 interaction may reduce intestinal inflammation, but at the cost of negatively interfering with hepatic immune responses. For instance, vedolizumab treatment might lead to reduced hepatic inflammation, yet paradoxically exacerbated steatosis in some cases [29]. Therefore, there is an unmet need for quality studies in carefully selected patients with IBD, focusing on the impact of biological agents on MASLD parameters, particularly those related to hepatic steatosis and fibrosis.

Given these considerations, the primary objective of this study was to assess the comparative six-month effects of infliximab (an anti-TNF agent) or vedolizumab (an anti-integrin agent) versus no biologic therapy on presumed hepatic steatosis in IBD patients. Secondary objectives included evaluating their relative effects on presumed hepatic fibrosis and other parameters related to hepatic metabolism and MASLD.

## 2. Results

### 2.1. Description of the Groups

**Figure 1:**
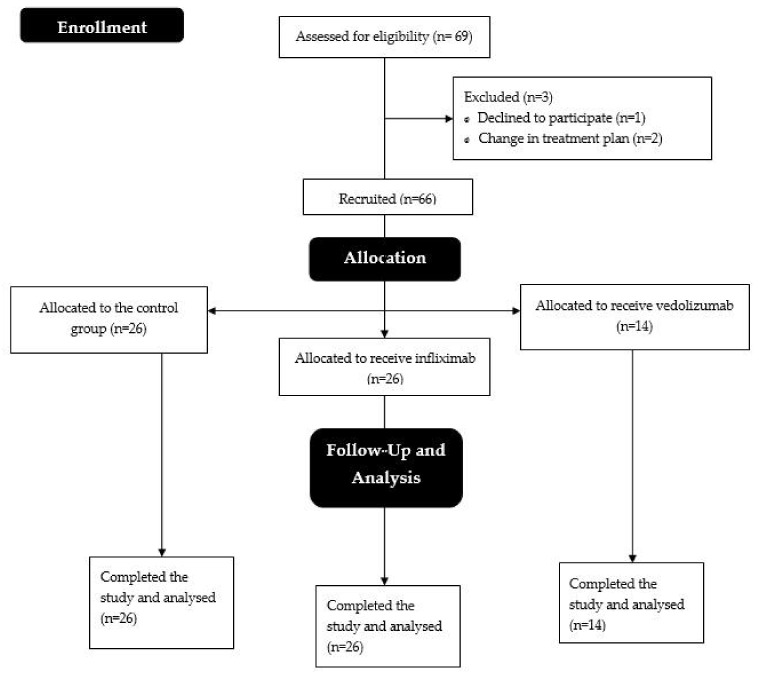
Study flowchart following the CONSORT template.

A total of sixty-six patients with IBD, who had not previously received biologics, were enrolled and allocated into three groups: control (n = 26), infliximab (n = 26), and vedolizumab (n = 14) (Figure 1). The primary characteristics of the patients in each group are outlined in Table 1. Among the 66 patients, 25 (37.9%) were women, and the male-to-female ratio was comparable across the groups (Table 1). The age in the infliximab and control groups was 38.0 ± 16.2 and 35.9 ± 15.3, respectively, whereas it was higher in the vedolizumab group (51.4 ± 17.9, *p* = 0.027). The duration of the IBD diagnosis did not differ significantly among groups. Half of the patients (33/66) had CD, with similar rates between groups (controls: 53.8%, infliximab: 46.2%, vedolizumab: 50%). However, patients assigned to infliximab had more active disease compared to the other groups, regardless of the subtype of IBD (Table 1).

In terms of the metabolic profile at baseline, BMI did not differ among groups, whereas waist circumference was larger in the infliximab compared to control group. The rates of arterial hypertension, type 2 diabetes mellitus (T2DM), cardiovascular diseases, and metabolic syndrome were not statistically different among groups.

At the baseline, 40.0% in the control group, 80.8% in the infliximab group, and 35.7% in the vedolizumab group had moderate/severe IBD, with the rates being higher in infliximab compared with the other two groups (*p* = 0.003). At the endpoint, no patient experienced a worsening of IBD; 34.6% of those in the control group, 50.0% in the infliximab group, and 21.4% in the vedolizumab group achieved downgrading of the inflammation from moderate/severe to mild/absent. Consequently, active disease (moderate/severe) was evident in 3.8%, 26.9%, and 14.3% of patients in the control, infliximab, and vedolizumab group, respectively, with the rates being lower in control compared with the other two groups (*p* = 0.018).

### 2.2. Primary Outcome

At the baseline, the rates of hepatic steatosis, assessed with the Hamaguchi score, were 23.1%, 26.9%, and 46.2% in the control, infliximab, and vedolizumab groups, respectively (*p* = 0.310). At the endpoint, these rates were 13.0%, 15.4%, and 15.4%, respectively, whilst without being statistically significant between or within groups (*p* = 0.765). At the baseline, FLI (controls = 36.2 ± 22.8, infliximab = 25.5 ± 23.2, vedolizumab = 40.2 ± 31.6; *p* = 0.151), HSI (controls = 34.4 ± 5.5, infliximab = 32.8 ± 9.4, vedolizumab = 34.5 ± 6.5; *p* = 0.678), and CAP (controls = 254.9 ± 69.1, infliximab = 235.4 ± 68.5, vedolizumab = 255.2 ± 52.1; *p* = 0.587) were not significantly different among groups. At the endpoint, FLI, HSI, and CAP were also similar among groups (FLI: controls = 35.5 ± 23.4, infliximab = 28.5 ± 28.5, vedolizumab = 39.9 ± 27.1; *p* = 0.216; HSI: controls = 34.1 ± 4.7, infliximab = 33.4 ± 7.6, vedolizumab = 35.6 ± 6.0; *p* = 0.575; CAP: controls = 231.4 ± 61.7, infliximab = 227.3 ± 42.7, vedolizumab = 256.3 ± 58.4; *p* = 0.456). Regarding the surrogate markers of hepatic steatosis (CAP, FLI, and HSI), no significant between or within group difference was similarly observed (Supplementary Table S1).

Regarding change in steatosis status (from baseline to endpoint) based on the Hamaguchi score, 12.0% of cases improved steatosis, 75% remained without steatosis, and 13.0% had persistent steatosis in the control group. In the infliximab group, the rates were 11.5%, 73.1%, and 15.4%, respectively, and in the vedolizumab group were 23.1%, 61.5%, and 15.4%, respectively. There were no significant differences between the three groups (*p* = 0.876). It should be highlighted that no patient in any group progressed from no steatosis (at baseline) to steatosis (at endpoint).

In binary logistic regression analysis (Table 2), the biologics (group), disease activity, disease duration, age, adiponectin, TNF, leptin, PIIINP, and TIMP-1 were sequentially entered as potential confounders, with the dependent variable being the presence of steatosis, based on the Hamaguchi score. At the baseline, the administration of infliximab or vedolizumab were not associated with steatosis. This pattern did not change, when other potential confounders were added to the model. Disease duration was marginally associated with steatosis independently from treatment and disease activity, which, however, did not remain robust when age was added to the model. Similarly, at the endpoint, biologics were not associated with steatosis (Table 3). Disease duration was not independently associated with steatosis at the endpoint.

Considering the 6-month dynamic effect of IBD treatment on some of the potential confounders, binary logistic regression was repeated with dependent variable the change in steatosis status “no steatosis” (including patients that remained without steatosis and those that regress steatosis) vs. steatosis (including patients with persistent steatosis), and potential confounders the biologics (group), the change in IBD activity, disease duration, age, and changes in adiponectin, TNF, leptin, PIINP, and/or TIMP-1 (endpoint minus baseline). No significant associations were observed between the dependent variable and the biologics or any other of the included variables (Supplementary Table S2). In subgroup analysis (separately for patients with CD or UC), no significant association between change in steatosis status and biologics or other potential confounders was observed (Supplementary Tables S3 and S4, respectively).

### 2.3. Secondary Outcomes

**Figure 2:**
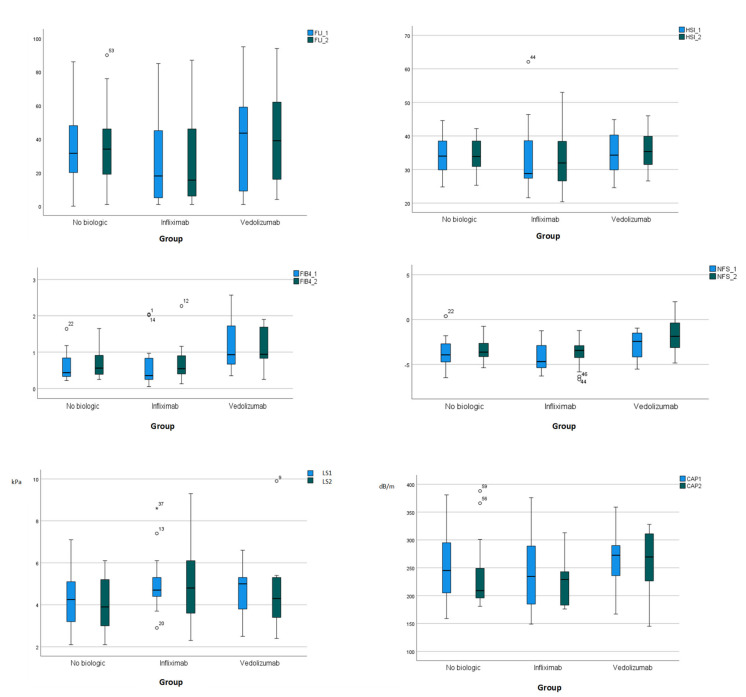
Box plots representing FLI, HSI, FIB-4, NFS, LS (kPa), and CAP (dB/m) values at baseline (cyan color) and endpoint (blue black color). CAP, controlled attenuation parameter; FLI, fatty liver index; FIB-4, fibrosis-4 index; HSI, hepatic steatosis index.

Based on LS by transient elastography or SWE, the majority of patients had no hepatic fibrosis at baseline. Furthermore, LS (by transient elastography) did not provide significant differences between or within groups or in biologic × time interactions (Supplementary Table S1); similarly, LS by SWE was not significantly different between groups at the endpoint (*p* = 0.476). However, FIB-4 significantly increased within infliximab and control group, and NFS within all groups after treatment; however, no significant trend was observed in biologic × time interactions. Figure 2 illustrates FLI, HSI, FIB-4, NFS, LS, and CAP at the baseline and the endpoint.

**Figure 3:**
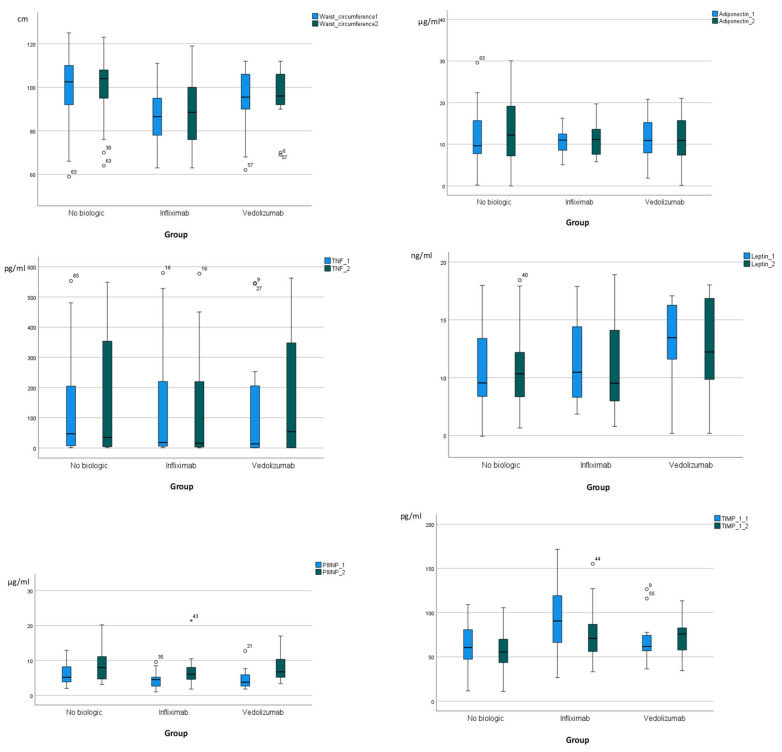
Box plots representing waist circumference (cm), adiponectin (μg/mL), TNF (pg/mL), leptin (ng/mL), PIIINP (μg/mL), TIMP-1 (ng/mL) at baseline (cyan color) and endpoint (blue black color). PIIINP, N-terminal propeptide of procollagen type III; TIMP-1.

The metabolic parameters—including BMI, triglycerides, glucose, insulin, and HOMA-IR—did not demonstrate any significant trends in the biologic × time interactions. However, waist circumference exhibited a trend in the biologic × time interactions after adjusting for changes in disease activity (model 1) and changes in disease activity plus age (model 2); this trend was primarily driven by an increase in waist circumference in the control group at the endpoint. PIINP concentrations were increased within all groups at the endpoint; however, they were without significant trend in biologic × time interactions. TIMP-1 was decreased within the infliximab group, and was also without significant trend in biologic × time interactions (Supplementary Table S1). No significant trends were observed in adiponectin, TNF, and leptin. Figure 3 illustrates waist circumference, adiponectin, TNF, leptin, PIIINP, TIMP-1 at the baseline and the endpoint. No significant trend in the biologic × time interactions was also observed in other variables (white blood cells, hemoglobulin, platelets, albumin, CRP) associated with MASLD and/or IBD.

Furthermore, the subgroup analysis, performed separately for patients with CD (Supplementary Table S5) and UC (Supplementary Table S6) did not reveal significant trends in biologic × time interactions, apart for albumin in UC; this significant biologic × time interactions was driven by an increase in albumin with the infliximab and vedolizumab groups, but not in the control group (Supplementary Table S6). Due to the small number of patients per group, we did not perform an adjustment for potential confounders in the subgroup analyses.

## 3. Discussion

To the best of our knowledge, this prospective, non-randomized, open-label study is the first of its kind designed to investigate the impact of biologics on hepatic steatosis, in previously naïve for biologics patients with an established diagnosis of IBD, who started treatment with infliximab or vedolizumab, compared to controls (patients not receiving biologics). No difference was shown in presumed steatosis between groups at the baseline or the endpoint. The duration of the IBD was positively associated with steatosis at the baseline; however, this association did not remain robust when age, an established associate of steatosis, was added to the model (Table 2). Interestingly, neither biologics nor the 6-month change in other potential confounders were associated with changes in steatosis status from the baseline to the endpoint (Supplementary Table S2). Similarly, no significant impact of biologics was shown on the other surrogate indices of steatosis (CAP, FLI and HSI; Supplementary Table S1). Regarding fibrosis, biologics have no effect on LS; however, the non-invasive indices FIB-4 and NFS, and PIIINP, the last used as a biomarker of increased collagen III synthesis, increased within all groups (apart from FIB-4 in the vedolizumab group), without, however, a specific trend in biologic × time interactions. Furthermore, the observed changes within groups may be statistically significant, but they seem to be of low magnitude to be clinically significant. We have no solid explanation for the discrepancy of the effects of biologics on the indices of hepatic fibrosis and LS. A plausible hypothesis may be the diminishing effect of both infliximab and vedolizumab on platelet count (Supplementary Table S1), which is included in the algorithms of both FIB-4 and NFS. Partial changes within BMI (infliximab group), AST (infliximab group), and ALT (vedolizumab group) after treatment may also account for the observed increase in FIB-4 and NFS. This occur despite the increasing effect of both infliximab and vedolizumab on albumin (Supplementary Table S1), which is also included in the algorithm of NFS. Whether changes in hepatic fibrosis indices may be an early event which is followed by a later increase in LS needs studies that will be focused on this aim and be probably of a longer duration.

Prior to our trial, the evidence on the effect of biologics on MASLD among patients with IBD was limited and indirect, with most studies not showing an association between biologics and MASLD prevalence or severity, which is largely confirmed by our findings [17,20,30,31]. However, one retrospective cross-sectional study indicated that IBD patients not receiving anti-TNF treatment had higher rates of hepatic steatosis (assessed through abdominal imaging) compared to those undergoing anti-TNF therapy [27]. In another case-control study, anti-TNF treatment was inversely associated with abnormal liver function tests in patients with IBD [32]. Regarding vedolizumab, a retrospective cohort study revealed that patients with IBD and CAP-defined hepatic steatosis underwent therapies with vedolizumab more frequently (16.7%) compared to those without steatosis (1.8%), implying a potentially adverse effect of vedolizumab on steatosis, although the design of this study cannot show causality [20]. Moreover, in a cross-sectional retrospective cohort, the use of vedolizumab was also associated with steatosis [6]. The different design of different studies, including the observational and retrospective nature of most of them, as well as population differences, may partly have led to different results between them and our study, which showed no effect of infliximab or vedolizumab on presumed hepatic steatosis after a 6-month treatment. These findings were largely confirmed in the subgroup analyses, separately for patients with CD and UC.

The remission of IBD activity has been supported to lead to weight gain upon achieving clinical response, thereby generating the substrate for MASLD development [7,33,34]. More specifically, disease remission predisposes to increased nutritional intake and normalization in the absorption of nutrients, possibly promoting obesity, metabolic dysfunction, and MASLD [7]. In our study, an increase in waist circumference was observed in the control group, but not in the infliximab or vedolizumab groups (Supplementary Table S1). This may imply that infliximab and vedolizumab had no effect on central adiposity, but also confirmed that waist circumference was increased in the control group, in which greater rates of remission or mild disease were observed at the endpoint.

In this study, no specific trend of leptin, adiponectin or TNF was shown, although all them have been highly associated with MASLD and its severity [35,36,37]. Furthermore, no compensatory increase of TNF was observed in the infliximab group, owing to the blockade of TNF by the infliximab. Similarly, no effect was observed in insulin resistance, assessed with the HOMA-IR index, glucose, or liver function tests. All these are in line with the observed lack of any response to hepatic steatosis after treatment with both biologics. PIIINP and TIMP-1 have been involved in the fibrogenesis, being potentially predictors of hepatic fibrosis. Indeed, PIIINP concentrations seem to increase in response to collagen deposition during fibrosis [38], and TIMP-1 has been implicated in the progression to hepatic fibrosis [39,40]. Although no biologic × time interactions was shown in our study, PIIINP was increased within all groups, whereas TIMP-1 was decreased only within the infliximab group after treatment (Supplementary Table S1). However, no secure conclusion can be made on this seemingly paradox, especially for the infliximab group.

Despite its originality, this study has certain limitations. First, randomization was not implemented, primarily due to ethical considerations surrounding the treatment of patients with moderate-to-severe IBD; leaving patients with active inflammation untreated or undertreated for six months could impact on IBD activity and prognosis, thus raising ethical considerations; however, the lack of randomization and the selection of medication based on good clinical practice increase the risk of selection bias. Second, a placebo was not utilized; given the different formulations of infliximab and vedolizumab, a double placebo would be necessary for the control group along with a single placebo for both the infliximab and vedolizumab groups, which could introduce methodological challenges, particularly with the parenteral administration routes of these drugs. These circumstances led to an open-label design, which, however, may have led to potential performance bias. To partly overcome this limitation, the clinical management and follow-up care for the IBD were determined by an independent physician, not involved in our study. Another source of potential selection bias arises from the study’s setting in a tertiary hospital, where patients with more severe IBD activity are often referred. Fourth, hepatic steatosis at baseline was not an inclusion criterion, which may potentially have impacted the results of the study; however, this was not a prespecified criterion, because it was initially largely unknown whether infliximab and vedolizumab had beneficial or adverse effects on hepatic steatosis, so in this way we would have been able to evaluate their effects on both directions (improvement in those with steatosis at baseline and worsening in those without steatosis at baseline). Furthermore, the follow-up of six months could not predicate the longer-term effects of biologics on steatosis and fibrosis, which remain to be shown in future studies. Moreover, steatosis and fibrosis were not evaluated with the gold standard method (histological confirmation), because double liver biopsy within six months meets certain ethical considerations, especially in patients with IBD, who have, by definition, a long history of multiple medical tests and procedures. Finally, subgroup analyses may be underpowered, which is why we refrained from extensive adjustments for potential confounders; nonetheless, these analyses largely supported the findings of the main investigation.

## 4. Materials and Methods

### 4.1. Study Design

This was a prospective, non-randomized, open-label, controlled trial, comprised from three arms: two biologic groups (infliximab or vedolizumab) and one control group, in which patients with IBD not starting treatment with biologics were assigned; these patients might have been treated with mesalamine, sulfasalazine, azathioprine, or budesonide, but not methotrexate, for IBD. Consecutive adult patients with established IBD, were recruited from the outpatient clinics of the Department of Gastroenterology, University General Hospital of Larissa, Greece, from December 2020 to March 2024. A predetermined protocol, complying with the ethical guidelines of the last revision of Declaration of Helsinki and conformed to the Good Clinical Practice Guidelines [41,42], was approved by the Scientific Committee of the University General Hospital of Larissa, Greece (Ref. No: 15/10/16-07-20) and by the Research Bioethics Committee of the School of Medicine, Aristotle University of Thessaloniki, Greece (Ref. No: 3.312/22.12.2020). All patients were provided a signed informed consent and anonymity was ensured.

### 4.2. Population

Inclusion criteria were: (1) patients ≥ 18 years; (2) established diagnosis of CD or UC; (3) no previous treatment with biologics (treatment naïve for biologics); (4) clinical decision to receive monotherapy with infliximab or vedolizumab, or remain without therapy with biologics, based on specific criteria for controlling IBD and in the setting of good clinical practice [43,44].

Exclusion criteria were: (1) denial to participate in the study or to sign the informed consent; (2) alcohol consumption ≥ 20 g/day; (3) diagnosis of other liver diseases (e.g., viral hepatitis, autoimmune hepatitis, primary sclerosing cholangitis, primary biliary cholangitis and variants, drug-induced liver injury, hemochromatosis, Wilson’s disease, α1-antitrypsin deficiency); (4) any malignant disease; (5) lipodystrophy; (6) history of bariatric/metabolic surgery and small bowel resection; (7) pregnancy; (8) use of medications associated with drug-induced liver injury (e.g., methotrexate, tamoxifen, amiodarone, aloperidin, hormone replacement therapy, contraceptives, anabolic steroids, any medication against tuberculosis, epilepsy, or viruses); (9) use of medications potentially associated with improvement in MASLD (e.g., vitamin E, pioglitazone, insulin, sulfonylureas, glucagon-like peptide-1 receptor agonists, sodium-glucose co-transporter-2 inhibitor, orlistat, ursodeoxycholic acid, spironolactone).

### 4.3. Methods

Patients were enrolled at the initiation of biologic therapy (baseline) and were re-evaluated six months later (endpoint). The clinical management and follow-up care for the IBD were determined by an independent physician and were not influenced by the study protocol. The routine for infliximab included intravenous injection at a dosage of 5 mg/kg at weeks 0, 2, 6, and then every 8 weeks; dose adjustment or shortening of injection intervals were decided per case depending on the disease activity by the above-mentioned independent physician. Regarding vedolizumab, injection intervals were the same as for infliximab, but a stable dose of 300 mg was used per injection [43,44]. At baseline and endpoint visits, a complete physical examination was performed and a predefined form was completed with information on medical history, demographics, and comorbidities. All patients were advised to follow a dietary plan based on the European Society for Clinical Nutrition and Metabolism (ESPEN) guidelines for patients with IBD [45].

Data on IBD phenotype, disease activity, and prior medications were collected, and endoscopic examinations were performed prior to the baseline visit. The Montreal classification, Crohn’s disease activity index (CDAI) and simple endoscopic score for Crohn’s disease (SES-CD) were used for the assessment of CD, and the Mayo score and the Montreal classification were used for assessment of UC; the validated thresholds to define disease activity and extent were adopted for each score [46,47,48]. Additionally, the patients were evaluated for parameters associated with MASLD: systolic and diastolic blood pressure, weight, height, and waist circumference were measured, and body mass index (BMI) was calculated [weight(kg)/height(m)^2^]. At the baseline and endpoint visits, morning blood sample was drawn after overnight fasting, for the evaluation of complete blood count, biochemical tests (glucose, albumin, aspartate aminotransferase [AST], alanine aminotransferase [ALT], alkaline phosphatase, gamma-glutamyl transpeptidase (GGT), triglycerides, insulin), and C-reactive protein (CRP). Serum samples, after centrifuging, were also separated and stored at −80 °C. After the completion of the study, frozen serum samples from baseline and endpoint were sent to the Laboratory of the Department of Immunology & Histocompatibility (University of Thessaly, Larissa, Greece) for the measurement of adiponectin, TNF, leptin, N-terminal propeptide of procollagen type III (PIIINP), and tissue inhibitor of metalloproteinase-1 (TIMP-1), using commercially available ELISA kits (FineTest, Wuhan Fine Biotech, China and AssayGenie Ltd., Dublin, Ireland). The characteristics of the assays were as follows: adiponectin (EH2593; intra-assay coefficient of variation (CV) < 8%, inter-assay CV < 10%, sensitivity: 0.938 ng/mL), TNF (EH0302-HS; intra-assay CV < 8%, inter-assay CV < 10%, sensitivity: 1.875 pg/mL), leptin (EH0217; intra-assay CV < 8%, inter-assay CV < 10%, sensitivity: 9.375 pg/mL), PIIINP (EH0960-HS; intra-assay CV < 8%, inter-assay CV < 10%, sensitivity: 18.75 pg/mL), and TIMP-1 (HUES01451; intra-assay CV < 6%, inter-assay CV < 6%, sensitivity: 90 pg/mL). Dilutions were performed, as indicated, guided by the respective standard curves, so that the results fell withing their linear part. Within the same assay, the samples were run in duplicates.

Semi-quantitative assessment of hepatic steatosis was performed using transabdominal ultrasonography based on the Hamaguchi score [49]. This calculates a total score after evaluating and grading three parameters; the hepatic echogenicity assessing the hepatorenal contrast (in the mid-axillary line), the attenuation depth and the vascular blurring. A total score ≥ 2 provides sensitivity of 91.7% and specificity of 100.0% for hepatic steatosis [49]. Transient elastography (Fibroscan™, Echosens, Paris, France), provided by the Department of Medicine and Research Laboratory of Internal Medicine [(National Expertise Center of Greece in Autoimmune Liver Diseases, General University Hospital of Larissa, Greece; Full Member of the European Reference Network on Hepatological Diseases (ERN RARE-LIVER)], was also used for the evaluation of presumed hepatic steatosis, assessed with the CAP, and presumed hepatic fibrosis, evaluated with liver stiffness (LS). LS, accounting for the presumed hepatic fibrosis, was also evaluated using 2D-shear wave elastography (2D-SWE; LOGIQ™ S8 R3.2.2.-XDclear, General Electric, Boston, MA, USA), provided by the Department of Radiology of the University General Hospital of Larissa.

The calculation of validated non-invasive indices was also used for the assessment of insulin resistance (IR), hepatic steatosis and fibrosis: homeostasis model assessment—insulin resistance (HOMA-IR) index for IR, the fatty liver index (FLI) and the hepatic steatosis index (HSI) for hepatic steatosis, and the fibrosis-4 index (FIB-4) and the NAFLD fibrosis score (NFS) for hepatic fibrosis [50,51,52,53,54].

### 4.4. Outcomes and Definitions

The primary endpoint of this study was the evaluation of the comparative six-month effect of infliximab or vedolizumab vs. no biologic on hepatic steatosis in patients with IBD, as assessed with the Hamaguchi score (<2: no steatosis; ≥2: steatosis), whereas CAP and non-invasive indices of steatosis were used as complementary tools. To dynamically evaluate the effects of interventions on steatosis, we assessed changes in steatosis status—determined by the Hamaguchi score—six months post-treatment. The potential outcomes were as follows: (1) sustained absence of steatosis; (2) regression of steatosis (transitioning from steatosis at baseline to no steatosis at endpoint); (3) persistent steatosis; and (4) progression to steatosis (transitioning from no steatosis at baseline to steatosis at endpoint).

Secondary objectives included the evaluation of the comparative six-month effects of infliximab or vedolizumab vs. no biologic treatment on IBD activity, liver function tests, metabolic parameters associated with MASLD, and presumed hepatic fibrosis (LS, FIB-4 and NAFLD fibrosis score) in patients with IBD.

### 4.5. Sample Size Calculation

A power analysis was conducted a priori using G-Power 3.1.9.6 for Mac OS (Heinrich-Heine Universität, Düsseldorf, Germany). Since there were no relevant studies (at the time of the initiation of this study) in the literature to support the sample size calculation, we chose the moderate effect size of 0.25 for the primary endpoint and a type α error probability of 0.05. These assumptions led to the requirement of a total sample size of 66 patients to achieve power of 95% [55].

### 4.6. Statistical Analysis

Data are presented as mean ± standard deviation (SD) for continuous variables or numbers and/or frequencies for categorical variables. The Kolmogorov–Smirnov test was used to evaluate the normality of distributions. Repeated measures ANOVA 2 × 2 (3 groups × 2 time points) was used to evaluate the overall tendency of group × time interactions. In case of statistically significant trend, post hoc pairwise comparisons were performed with Tukey’s correction for multiple comparisons. For within group comparisons (baseline to endpoint), a paired T-test or Wilcoxon signed-rank test was used for normally and non-normally distributed continuous variables, respectively. An independent T-test or Mann–Whitney test was used for the comparisons between groups (separately at baseline and at the endpoint) for normally and non-normally distributed continuous variables, respectively. A chi-square test or Fischer’s exact test was used for the comparisons of categorical variables between groups. Repeated measured analysis of covariance (ANCOVA) was used to adjust group × time interactions for potential confounders, which were: disease activity (model 1); disease activity and age (model 2); disease activity, age, and waist circumference (model 3); disease activity, age, waist circumference, and adiponectin concentrations (model 4); disease activity, age, waist circumference, and TNF concentrations (model 5); disease activity, age, waist circumference, and leptin concentrations (model 6); disease activity, age, waist circumference, and PIIINP concentrations (model 7); disease activity, age, waist circumference, and TIMP-1 concentrations (model 8). For dichotomous variables (e.g., Hamaguchi score or change in Hamaguchi score), binary regression analysis was used to adjust the associations for potential confounding factors, and the results are presented as odds ratios with 95% confidence interval (95%CI). The Hosmer–Lemeshow test was used to check the goodness-of-fit of the multivariable regression model. To investigate the potentially confounding effect of changes in IBD activity, adiponectin, TNF, leptin, PIIIMP, and TIMP-1 on the change in the dependent variable (change in Hamaguchi score), new variables were created reflecting the changes from baseline to six months (endpoint minus baseline values). As sensitivity analysis, we repeated most of the abovementioned analyses separately in CD and UC subgroups. A two-sided *p*-value < 0.05 was considered statistically significant in all tests. Statistical analysis was performed with SPSS for Windows, version 26 (IBM Corporation, Chicago, IL, USA).

## 5. Conclusions

In conclusion, this study did not find any positive or negative effects of infliximab or vedolizumab compared to no biologic treatment on presumed hepatic steatosis in biologic-naïve patients with IBD. Although no impact on LS was observed, the slight but significant increase in non-invasive indices of fibrosis and PIIINP suggests the need for further long-term studies with potentially larger sample sizes.

## Figures and Tables

**Table 1:** Main characteristics of the patients per group.

		Controls (n = 26)	Infliximab (n = 26)	Vedolizumab (n = 14)	*p*-Value for Trend
Demographics	Sex (Women)	11 (42.3%)	8 (30.8%)	6 (42.9%)	0.630
	Age (Years)	35.9 ± 15.3	38.0 ± 16.2	51.4 ± 17.9 ^a,b^	0.027
	Smoking (Current)	1 (3.8%)	10 (38.5%)	3 (21.4%)	0.011
	BMI (kg/m^2^)	24.9 ± 4.2	23.4 ± 4.9	26.5 ± 4.3	0.111
	Waist circumference (cm)	87.0 ± 13.8	99.1 ± 15.4 ^a^	94.9 ± 19.9	0.025
IBD diagnosis	Crohn’s disease	14 (53.8%)	12 (46.2%)	7 (50.0%)	0.857
Disease activity	Disease duration (years)	6.9 ± 8.6	6.0 ± 8.4	11.6 ± 11.8	0.084
	Active disease *	10 (40.0%)	21 (80.8%)	5 (35.7%)	0.003
	CDAI	47.8 ± 140.8	165.4 ± 62.8 ^a^	80.4 ± 60.1	0.024
	Mayo score	5.1 ± 3.7	9.64 ± 1.6 ^a^	6.9 ± 3.1	0.002
Metabolic comorbidities	Arterial hypertension; N (%)	2 (7.7%)	1 (3.8%)	3 (21.4%)	0.173
	Type 2 diabetes mellitus; N (%)	1 (3.8%)	0 (0.0%)	2 (14.3%)	0.115
	Cardiovascular disease; N (%)	0 (0.0%)	1 (3.8%)	0 (0.0%)	0.458
	Metabolic syndrome; N (%)	2 (7.7%)	1 (3.8%)	3 (21.4%)	0.173
Hamaguchi score	Steatosis; N (%)	6 (23.1%)	7 (26.9%)	6 (46.2%)	0.310
Transient elastography; steatosis	CAP (dB/m)	254.9 ± 69.1	235.4 ± 68.5	255.2 ± 52.1	0.587
Non-invasive hepatic steatosis indices	FLI < 30; N (%)	12 (46.2%)	16 (64.0%)	5 (38.5%)	0.507
	HSI < 30; N (%)	8 (30.8%)	13 (50.0%)	4 (28.6%)	0.069
SWE; liver stiffness	F0; N (%)	22 (88.0%)	25 (96.2%)	12 (92.3%)	0.172
	F1; N (%)	3 (12.0%)	1 (3.8%)	0 (0.0%)	
	F4; N (%)	0 (0.0%)	0 (0.0%)	1 (7.7%)	
Transient elastography; liver stiffness	F0; N (%)	20 (83.3%)	23 (92.0%)	13 (100.0%)	0.144
	F1; N (%)	4 (16.7%)	0 (0.0%)	0 (0.0%)	
	F2; N (%)	0 (0.0%)	1 (4.0%)	0 (0.0%)	
	F3; N (%)	0 (0.0%)	1 (4.0%)	0 (0.0%)	
Non-invasive hepatic fibrosis indices	FIB-4 < 1.3; N (%)	25 (96.2%)	24 (92.3%)	9 (64.3%)	0.009
	NFS < −1.455; N (%)	25 (96.2%)	24 (92.3%)	10 (71.4%)	0.044

Abbreviations: BMI, body mass index; CAP, controlled attenuation parameter; CD, Crohn’s disease; CDAI, Crohn’s disease activity index; FIB-4, Fibrosis-4 index; FLI, Fatty Liver Index; HSI, Hepatic steatosis index; IBD, inflammatory bowel disease; NFS, NAFLD fibrosis score; SWE, shear-waive elastography; * Based on CDAI index and Mayo score for CD and UC, respectively. ^a^: Compared to controls, ^b^: Compared to infliximab (pairwise comparisons after Tukey’s correction).

**Table 2:** Binary logistic regression analysis sequentially evaluating the potential impact of biologics (group), disease activity, disease duration, age, adiponectin, TNF, leptin, PIIINP, and/or TIMP-1 on the presence of steatosis, based on the Hamaguchi scale at baseline.

	Model 1		Model 2		Model 3		Model 4		Model 5		Model 6		Model 7		Model 8		Model 9	
	Odds Ratio	95% CI	Odds Ratio	95% CI	Odds Ratio	95% CI	Odds Ratio	95% CI	Odds Ratio	95% CI	Odds Ratio	95% CI	Odds Ratio	95% CI	Odds Ratio	95% CI	Odds Ratio	95% CI
Infliximab ^1^	1.23	0.35–4.32	1.44	0.37–5.73	1.58	0.37–6.72	1.48	0.34–6.39	1.44	0.33–6.39	1.38	0.31–6.09	1.51	0.35–6.59	1.47	0.34–6.39	1.42	0.30–6.75
Vedolizumab ^1^	2.86	0.69–11.84	2.73	0.65–11.47	2.42	0.52–11.35	2.07	0.41–10.37	2.04	0.40–10.29	1.64	0.31–8.73	1.76	0.24–9.24	2.19	0.43–11.25	2.04	0.40–10.36
Moderate–severe disease activity ^2^			0.59	0.18–2.00	0.65	0.18–2.34	0.68	0.19–2.44	0.68	0.18–2.52	0.66	0.18--2.42	0.62	0.17–2.28	0.67	0.18–2.41	0.67	0.18–2.44
Disease duration					1.09	1.02–1.16	1.08	0.99–1.16	1.07	0.99–1.16	1.08	0.99–1.15	1.08	1.00–1.16	1.08	0.99–1.16	1.08	0.99–1.16
Age							1.02	0.97–1.06	1.04	0.97–1.06	1.02	0.98–1.07	1.01	0.97–1.06	1.01	0.97–1.06	1.01	0.97–1.06
Adiponectin									0.99	0.86–1.13								
TNF											1.00	0.99–1.01						
Leptin													1.07	0.89–1.29				
PIIINP															0.98	0.89–1.08		
TIMP-1																	1.00	0.98–1.02

Abbreviations: CI, confidence interval; PIIINP, N-terminal propeptide of procollagen type III; TIMP-1, tissue inhibitors of metalloproteinase 1; TNF, tumor necrosis factor. Model 1: unadjusted (crude model); Model 2: adjusted for disease activity at baseline; Model 3: adjusted for disease activity and disease duration at baseline; Model 4: adjusted for disease activity, disease duration, and age at baseline; Model 5: adjusted for disease activity, disease duration, age, and adiponectin at baseline; Model 6: adjusted for disease activity, disease duration, age, and TNF at baseline; Model 7: adjusted for disease activity, disease duration, age, and leptin at baseline; Model 8: adjusted for disease activity, disease duration, age, and PIIINP at baseline; Model 9: adjusted for disease activity, disease duration, age, and TIMP-1 at baseline. ^1^: Compared to control group (reference), ^2^: compared to IBD remission or mild disease activity.

**Table 3:** Binary logistic regression analysis sequentially evaluating the potential impact of biologics (group), disease activity, disease duration, age, adiponectin, TNF, leptin, PIIINP, and/or TIMP-1 on the presence of steatosis, based on the Hamaguchi scale at the endpoint.

	Model 1		Model 2		Model 3		Model 4		Model 5		Model 6		Model 7		Model 8		Model 9	
	Odds Ratio	95% CI	Odds Ratio	95% CI	Odds Ratio	95% CI	Odds Ratio	95% CI	Odds Ratio	95% CI	Odds Ratio	95% CI	Odds Ratio	95% CI	Odds Ratio	95% CI	Odds Ratio	95% CI
Infliximab ^1^	1.27	0.25–6.38	2.45	0.44–13.79	2.57	0.44–15.03	2.57	0.44–15.00	2.61	0.44–15.49	2.44	0.41–14.56	2.61	0.44–15.42	2.71	0.43–16.89	3.23	0.48–21.72
Vedolizumab ^1^	1.91	0.33–11.08	2.10	0.36–12.37	1.61	0.25–10.34	1.58	0.34–10.45	1.59	0.24–10.71	1.29	0.19–8.67	1.58	0.24–10.51	1.57	0.22–11.34	2.07	0.26–16.44
Moderate–severe disease activity ^2^			0.27	0.03–2.69	0.26	0.03–2.79	0.26	0.02–2.78	0.26	0.02–2.76	0.26	0.02–2.92	0.24	0.02–2.90	0.20	0.02–2.39	0.27	0.02–3.05
Disease duration					1.04	0.97–1.12	1.04	0.96–1.13	1.04	0.96–1.13	1.04	0.95–1.14	1.04	0.95–1.14	1.04	0.95–1.14	1.04	0.96–1.14
Age							1.00	0.95–1.06	1.00	0.95–1.06	1.01	0.96–1.07	1.01	0.95–1.06	0.99	0.94–1.06	1.00	0.95–1.06
Adiponectin									1.01	0.89–1.14								
TNF											1.00	0.99–1.01						
Leptin													0.98	0.81–1.20				
PIIINP															0.88	0.69–1.11		
TIMP-1																	0.99	0.96–1.02

Abbreviations: CI, confidence interval; PIIINP, N-terminal propeptide of procollagen type III; TIMP-1, tissue inhibitors of metalloproteinase 1; TNF, tumor necrosis factor. Model 1: unadjusted (crude model); Model 2: adjusted for disease activity at baseline; Model 3: adjusted for disease activity and disease duration at baseline; Model 4: adjusted for disease activity, disease duration, and age at baseline; Model 5: adjusted for disease activity, disease duration, age, and adiponectin at baseline; Model 6: adjusted for disease activity, disease duration, age and TNF at baseline; Model 7: adjusted for disease activity, disease duration, age, and leptin at baseline; Model 8: adjusted for disease activity, disease duration, age, and PIIINP at baseline; Model 9: adjusted for disease activity, disease duration, age, and TIMP-1 at baseline. ^1^: Compared to control group (reference), ^2^: compared to IBD remission or mild disease activity.

## Data Availability

The original contributions presented in the study are included in the article/Supplementary Materials, further inquiries can be directed to the corresponding author.

## Supplementary Materials

The following supporting information can be downloaded at: https://www.mdpi.com/article/10.3390/ph17111432/s1, Table S1. Comparative baseline and 6-months data of the three groups on variables related to hepatic steatosis and fibrosis and associated parameters. Table S2. Binary logistic regression analysis sequentially evaluating the potential impact of biologics (group), change in IBD activity, disease duration, age, and changes in adiponectin, TNF, leptin, PIIINP and/or TIMP-1 on the change in steatosis status. Table S3. Binary logistic regression analysis sequentially evaluating the potential impact of biologics (group), change in IBD activity, disease duration, age, and changes in adiponectin, TNF, leptin, PIIINP and/or TIMP-1 on the change in steatosis status, separately in patients with Crohn’s disease. Table S4. Binary logistic regression analysis sequentially evaluating the potential impact of biologics (group), change in IBD activity, disease duration, age, and changes in adiponectin, TNF, leptin, PIIINP and/or TIMP-1 on the change in steatosis status, separately in patients with ulcerative colitis. Table S5. Comparative baseline and 6-months data of the three groups on variables related to hepatic steatosis and fibrosis and associated parameters separately in patients with Crohn’s disease. Table S6. Comparative baseline and 6-months data of the three groups on variables related to hepatic steatosis and fibrosis and associated parameters separately in patients with ulcerative colitis.
