# Supplementary material for: The Effect of Biologic Agents on Steatotic Liver Disease in Patients with Inflammatory Bowel Disease: A Prospective, Open-Label Comparative Trial
Source: Pharmaceuticals (Basel). 2024 Oct 25;17(11):1432. doi: 10.3390/ph17111432 (PMC11597268; doi:10.3390/ph17111432)
Supplement: Supplementary file 1 [file pharmaceuticals-17-01432-s001.zip › pharmaceuticals-3265790-supplementary.pdf]

Supplementary Table S1. Comparative baseline and 6-months data of the three groups on variables related to hepatic steatosis and fibrosis and associated parameters.

		Timepoint	Controls (n=26)	Infliximab (n=26)	Vedolizumab (n=14)	p-value (between groups)	p-value for trend	p-value for trend model 1	p-value for trend model 2	p-value for trend model 3	p-value for trend model 4	p-value for trend model 5	p-value for trend model 6	p-value for trend model 7	p-value for trend model 8
Hepatic steatosis related parameters	Body mass index (kg/m^2)	Baseline	24.9 ±4.2`	23.4 ±4.9	26.5 ±4.3	0.111	0.098	0.394	0.263	0.461	0.471	0.374	0.412	0.542	0.464
		Month 6	24.9 ±3.9	24.5 ±5.7	26.8 ±3.4	0.296									
	p-value (within groups)		0.991	0.010	0.481										
	Waist circumference (cm)	Baseline	87.0 ±13.8	99.1 ±15.4[a]	94.9±19.9	0.025	0.056	0.035	0.045	N/A	N/A	N/A	N/A	N/A	N/A
		Month 6	90.4 ±158	99.5 ±14.1	94.6 ±16.3	0.104									
	p-value (within groups)		0.004	0.597	0.865										
	Controlled attenuation parameter (dB/m)	Baseline	254.9 ±69.1	235.4 ±68.5	255.2 ±52.1	0.587	0.690	0.873	0.546	0.846	0.518	0.738	0.845	0.524	0.452
		Month 6	231.4 ±61.7	227.3 ±42.7	256.3 ±58.4	0.456									
	p-value (within groups)		0.054	0.281	0.677										
	Fatty liver index	Baseline	36.2 ±22.8	25.5 ±23.2	40.2 ±31.6	0.151	0.729	0.629	0.634	N/A	N/A	N/A	N/A	N/A	N/A
		Month 6	35.5 ±23.4	28.5 ±28.5	39.9 ±27.1	0.216									
	p-value (within groups)		0.809	0.915	0.730										
	Hepatic steatosis index	Baseline	34.4 ±5.5	32.8 ±9.4	34.5 ±6.5	0.678	0.538	0.549	0.347	0.248	0.457	0.448	0.533	0.455	0.516
		Month 6	34.05 ±4.7	33.39 ±7.6	35.6 ±6.0	0.575									

	p-value (within groups)		0.620	0.511	0.356										
Liver fibrosis related parameters	Liver stiffness using transient elastography (kPa)	Baseline	4.4 ±1.3	5.0 ±1.3	4.9 ±1.4	0.208	0.969	0.743	0.581	0.633	0.387	0.397	0.597	0.609	0.421
		Month 6	3.9 ±1.3	4.9 ±1.8	4.3 ±1.4	0.193									
	p-value (within groups)		0.322	0.625	0.404										
	FIB-4	Baseline	0.6 ±0.4	0.6 ±0.5	1.1 ±0.7[a,b]	0.002	0.970	0.992	0.995	0.996	0.825	0.897	0.980	0.830	0.635
		Month 6	0.7 ±0.3	0.7 ±0.4	1.3 ±0.9	0.071									
	p-value (within groups)		0.003	0.011	0.600										
	NFS	Baseline	-3.8 ±1.4	-4.5 ±2.4	-2.8 ±1.5[b]	0.035	0.208	0.257	0.155	0.252	0.231	0.221	0.307	0.277	0.147
		Month 6	-3.4 ±1.0	-3.7 ±1.4	-1.8 ±1.8[a,b]	0.005									
	p-value (within groups)		0.037	0.007	0.048										
Laboratory tests	White blood cells (M/µL)	Baseline	8.6 ±2.4	11.2 ±3.7[a]	7.5 ±2.5[b]	<0.001	0.096	0.178	0.265	0.259	0.491	0.312	0.249	0.264	0.907
		Month 6	7.3 ±2.1	8.7 ±2.9	7.1 ±7.7	0.049									
	p-value (within groups)		0.021	0.001	0.482										
	Hemoglobin (g/dL)	Baseline	13.0 ±1.9	12.3 ±2.1	13.0 ±1.2	0.348	0.389	0.418	0.386	0.553	0.710	0.130	0.506	0.453	0.841
		Month 6	13.2 ±1.7	13.2 ±1.2	13.3 ±1.1	0.922									
	p-value (within groups)		0.451	0.025	0.550										
	Platelets (K/µL)	Baseline	287.0 ±79.9	382.9 ±171.8[a]	314.6 ±67.1	0.015	0.062	0.087	0.072	0.120	0.098	0.180	0.119	0.113	0.130
		Month 6	267.2 ±65.1	306.4 ±94.3	265.4 ±67.4	0.138									

p-value (within groups)		0.025	0.002	0.016										
C-reactive protein (mg/dL)	Baseline	1.6 ±3.5	3.4 ±7.3	1.5 ±2.9	0.151	0.398	0.810	0.145	0.809	0.958	0.749	0.640	0.791	0.904
	Month 6	0.4 ±0.8	0.5 ±0.5	0.5 ±0.9	0.312									
p-value (within groups)		0.029	<0.001	0.152										
Glucose (mg/dL)	Baseline	86.4 ±11.1	85.8 ±21.4	95.4 ±20.5	0.236	0.340	0.421	0.614	0.573	0.779	0.673	0.776	0.702	0.730
	Month 6	84.8 ±8.7	87.5 ±14.8	87.7 ±36.6	0.826									
p-value (within groups)		0.542	0.581	0.753										
Albumin (g/dL)	Baseline	4.4 ±0.5	4.0 ±0.6	4.2 ±0.5	0.067	0.162	0.606	0.632	0.701	0.834	0.609	0.753	0.708	0.924
	Month 6	4.5 ±0.3	4.4 ±0.3	4.4 ±0.3	0.212									
p-value (within groups)		0.071	<0.001	0.013										
Aspartate aminotransferase (U/L)	Baseline	21.7 ±21.9	20.1 ±15.6	34.1 ±17.5	0.003	0.347	0.349	0.338	0.350	0.215	0.505	0.324	0.425	0.574
	Month 6	23.5 ±14.7	24.9 ±11.4	27.3 ±26.9	0.578									
p-value (within groups)		0.046	0.038	0.177										
Alanine aminotransferase (U/L)	Baseline	22.4 ±20.0	19.8 ±15.5	25.7 ±13.2	0.068	0.227	0.153	0.131	0.126	0.075	0.142	0.104	0.141	0.224
	Month 6	23.9 ±160.3	25.5 ±16.5	19.7 ±8.8	0.669									
p-value (within groups)		0.492	0.057	0.035										
Gamma-glutamyl transpeptidase (U/L)	Baseline	21.9 ±20.9	21.4 ±18.9	22.8 ±24.6	0.979	0.963	0.889	0.916	0.938	0.368	0.359	0.412	0.368	0.347
	Month 6	17.3 ±10.6	17.3 ±18.9	19.1 ±13.6	0.354									

	p-value (within groups)		0.485	0.044	0.859										
	Triglycerides (mg/dL)	Baseline	80.2 ±30.3	111.5 ±75.9	110.2 ±50.9	0.086	0.399	0.341	0.355	0.449	0.232	0.527	0.363	0.368	0.322
		Month 6	88.2 ±37.6	102.2 ±38.9	125.9 ±76.3	0.339									
	p-value (within groups)		0.213	0.778	0.382										
	Insulin (U/mL)	Baseline	15.1 ±9.3	18.2 ±17.3	13.8 ±23.8	0.069	0.342	0.654	0.749	0.801	0.790	0.931	0.684	0.730	0.853
		Month 6	12.5 ±6.9	12.4 ±9.4	18.7 ±16.3	0.334									
	p-value (within groups)		0.141	0.109	0.161										
	HOMA-IR	Baseline	3.3 ±2.2	4.4 ±5.8	3.7 ±6.7	0.227	0.955	0.981	0.810	0.699	0.605	0.675	0.684	0.678	0.600
		Month 6	2.7 ±1.6	3.3 ±3.9	4.6 ±3.9	0.340									
	p-value (within groups)		0.126	0.460	0.236										
	Adiponectin (μg/ml)	Baseline	11.8 ±6.5	10.7 ±2.8	11.4 ±5.1	0.956	0.593	0.644	0.598	0.950	N/A	0.516	0.422	0.473	0.422
		Month 6	13.6 ±8.4	11.2 ±4.3	11.7 ±5.9	0.811									
	p-value (within groups)		0.228	0.304	0.433										
Adipokines/cytokines and serological markers of fibrosis	TNF (pg/ml)	Baseline	135.8 ±168.1	134.7 ±183.6	120.9 ±196.3	0.557	0.389	0.399	0.436	0.347	0.448	N/A	0.310	0.359	0.628
		Month 6	161.3 ±209.4	148.9 ±192.9	188.5 ±227.8	0.906									
	p-value (within groups)		0.961	0.103	0.013										
	Leptin (ng/ml)	Baseline	10.6 ±3.6	11.2 ±3.4	12.8 ±3.8	0.167	0.840	0.915	0.536	0.593	0.539	0.717	N/A	0.697	0.664
		Month 6	10.9 ±3.6	11.0 ±3.9	12.7 ±4.3	0.324									
	p-value (within groups)		0.292	0.970	0.594										
		Baseline	5.9 ±2.8	5.9 ±7.5	7.0 ±9.8	0.179	0.580	0.610	0.556	0.466	0.578	0.402	0.453	N/A	0.569

PIIINP (μg/L)	Month 6	9.7 ±8.5	7.9 ±7.5	9.8 ±9.5	0.407									
p-value (within groups)		0.007	0.004	0.016										
TIMP-1 (ng/ml)	Baseline	63.6 ±26.4	92.3 ±36.7[a]	68.8 ±25.5	0.004	0.194	0.324	0.246	0.274	0.249	0.456	0.307	0.275	N/A
	Month 6	54.3 ±25.6	74.1 ±27.5[a]	73.9 ±19.3	0.011									
p-value (within groups)		0.241	0.044	0.371										

Data are presented as mean ± standard deviation.

Model 1: adjusted for change in disease activity; Model 2: adjusted for change in disease activity and age; Model 3: adjusted for change in disease activity, age and change in waist circumference; Model 4: adjusted for change in disease activity, age, change in waist circumference and change in adiponectin; Model 5: adjusted for change in disease activity, age, change in waist circumference and change in TNF; Model 6: adjusted for change in disease activity, age, change in waist circumference and change in leptin; Model 7: adjusted for change in disease activity, age, change in waist circumference and change in PIIINP; Model 8: adjusted for change in disease activity, age, change in waist circumference and change in TIMP-1 (repeated measures analysis of covariance [ANCOVA] 2 × 2).

a: Compared to controls, b: Compared to infliximab (pairwise comparisons after Tukey's correction for multiple comparisons).

Abbreviations: FIB-4, Fibrosis-4 index; HOMA-IR, Homeostasis Model Assessment of Insulin Resistance; NFS, NAFLD fibrosis score; PIIINP, N-terminal propeptide of procollagen type III; TIMP-1, tissue inhibitors of metalloproteinase 1; TNF, tumor necrosis factor.

Supplementary Table S2. Binary logistic regression analysis sequentially evaluating the potential impact of biologics (group), change in IBD activity, disease duration, age, and changes in adiponectin, TNF, leptin, PIIINP and/or TIMP-1 on the change in steatosis status.*

	**Model 1**		**Model 2**		**Model 3**		**Model 4**		**Model 5**		**Model 6**		**Model 7**		**Model 8**		**Model 9**	
	**Odds ratio**	**95% CI**	**Odds ratio**	**95% CI**	**Odds ratio**	**95% CI**	**Odds ratio**	**95% CI**	**Odds ratio**	**95% CI**	**Odds ratio**	**95% CI**	**Odds ratio**	**95% CI**	**Odds ratio**	**95% CI**	**Odds ratio**	**95% CI**
Infliximab[1]	1.27	0.25-6.38	2.29	0.40-13.08	2.41	0.41-14.25	2.4	0.41-14.17	2.39	0.40-14.28	2.60	0.42-16.31	2.69	0.43-17.08	2.39	0.39-14.81	2.37	0.39-14.49
Vedolizumab	1.91	0.33-11.08	2.04	0.34-12.22	1.61	0.25-10.47	1.57	0.24-10.42	1.75	0.25-12.41	1.46	0.22-9.59	1.40	0.20-9.67	1.39	0.19-9.78	2.01	0.27-14.95
Changed from IBD flare to remission[2]			1.14	0.25-5.31	1.22	0.26-5.78	1.27	0.25-6.44	1.67	0.29-9.67	1.41	0.28-7.16	1.15	0.22-5.93	1.15	0.22-5.95	1.07	0.19-5.72
Remained in IBD flare[2]			0.29	0.03-3.29	0.29	0.02-3.54	0.29	0.02-3.49	0.34	0.03-4.23	0.29	0.02-3.57	0.21	0.01-3.26	0.27	0.02-3.54	0.29	0.02-3.75
Disease duration					1.04	0.97-1.12	1.04	0.94-1.13	1.04	0.95-1.13	1.04	0.95-1.13	1.04	0.95-1.13	1.04	0.96-1.14	1.04	0.95-1.13
Age							1.01	0.95-1.06	1.00	0.95-1.06	1.00	0.95-1.06	1.01	0.95-1.07	1.01	0.95-1.06	1.00	0.95-1.06
Change in Adiponectin									1.07	0.92-1.26								
Change in TNF											1.00	0.99-1.01						
Change in Leptin													0.92	0.71-1.19				
Change in PIIINP															0.88	0.71-1.09		
Change in TIMP-1																	0.99	0.97-1.01

Abbreviations: CI, confidence interval; PIIINP, N-terminal propeptide of procollagen type III; TIMP-1, tissue inhibitors of metalloproteinase 1; TNF, tumor necrosis factor.

*: Changes in steatosis status (dependent variable) was defined as “no steatosis” (including patients that remained without steatosis and those that regress steatosis) vs. steatosis (including patients with persistent steatosis); changes in continuous variables were calculated as endpoint minus baseline values.

Model 1: Unadjusted (crude model); Model 2: adjusted for disease activity at baseline; Model 3: adjusted for disease activity and disease duration at baseline; Model 4: adjusted for disease activity, disease duration, and age at baseline; Model 5: adjusted for disease activity, disease duration, age and adiponectin at baseline; Model 6: adjusted for disease activity, disease duration, age and TNF at baseline; Model 7: adjusted for disease activity, disease duration, age and leptin at baseline; Model 8: adjusted for disease activity, disease duration, age and PIIINP at baseline; Model 9: adjusted for disease activity, disease duration, age and TIMP-1 at baseline.

1: compared to controls, 2: compared to those remained in remission-mild disease activity.

Supplementary Table S3. Binary logistic regression analysis sequentially evaluating the potential impact of biologics (group), change in IBD activity, disease duration, age, and changes in adiponectin, TNF, leptin, PIIINP and/or TIMP-1 on the change in steatosis status, separately in patients with Crohn's disease.*

	Model 1		Model 2		Model 3		Model 4	
	Odds ratio	95% CI	Odds ratio	95% CI	Odds ratio	95% CI	Odds ratio	95% CI
Infliximab[1]	1.09	0.06-19.63	1.59	0.08-29.80	2.87	0.10-81.69	2.85	0.09-81.81
Vedolizumab[1]	9.00	0.72-113.02	7.93	0.61-103.46	12.10	0.58-252.58	12.01	0.57-253.17
Changed from IBD flare to remission[2]			0.79	0.06-10.27	0.80	005-12.51	0.82	0.05-14.00
Remained in IBD flare[2]			0.00	0.00-0.00	0.00	0.00-0.00	0.00	0.00-0.00
Disease duration					1.12	0.97-1.28	1.12	0.96-1.29
Age							1.00	0.93-1.08

Abbreviations: CI, confidence interval; PIIINP, N-terminal propeptide of procollagen type III; TIMP-1, tissue inhibitors of metalloproteinase 1; TNF, tumor necrosis factor

*: Changes in steatosis status (dependent variable) was defined as "no steatosis" (including patients that remained without steatosis and those that regress steatosis) vs. steatosis (including patients with persistent steatosis); changes in continuous variables were calculated as endpoint minus baseline values.

Model 1: Unadjusted (crude model); Model 2: adjusted for disease activity at baseline; Model 3: adjusted for disease activity and disease duration at baseline; Model 4: adjusted for disease activity, disease duration, and age at baseline

1: compared to controls, 2: compared to those remained in remission-mild disease activity.

Supplementary Table S4. Binary logistic regression analysis sequentially evaluating the potential impact of biologics (group), change in IBD activity, disease duration, age, and changes in adiponectin, TNF, leptin, PIIINP and/or TIMP-1 on the change in steatosis status, separately in patients with ulcerative colitis.*

	**Model 1**		**Model 2**		**Model 3**		**Model 4**	
	**Odds ratio**	**95% CI**	**Odds ratio**	**95% CI**	**Odds ratio**	**95% CI**	**Odds ratio**	**95% CI**
Infliximab[1]	1.23	0.17-9.02	4.00	0.21-75.66	3.69	0.18-74.96	3.26	0.16-67.66
Vedolizumab[1]	0.00	0.00-0.00	0.00	0.00-0.00	0.00	0.00-0.00	0.00	0.00-0.00
Changed from IBD flare to remission[2]			1.00	0.05-22.18	1.14	0.05-27.45	0.05	0.05-46.22
Remained in IBD flare[2]			0.20	0.003-14.01	0.14	0.002-12.75	0.00	0.001-12.33
Disease duration					1.07	0.94-1.21	0.85	0.85-1.23
Age							0.91	0.91-1.19

Abbreviations: CI, confidence interval; PIIINP, N-terminal propeptide of procollagen type III;TIMP-1, tissue inhibitors of metalloproteinase 1; TNF, tumor necrosis factor

*: Changes in steatosis status (dependent variable) was defined as “no steatosis” (including patients that remained without steatosis and those that regress steatosis) vs. steatosis (including patients with persistent steatosis); changes in continuous variables were calculated as endpoint minus baseline values.

Model 1: Unadjusted (crude model); Model 2: adjusted for disease activity at baseline; Model 3: adjusted for disease activity and disease duration at baseline; Model 4: adjusted for disease activity, disease duration, and age at baseline

1: compared to controls, 2: compared to those remained in remission-mild disease activity.

Supplementary Table S5. Comparative baseline and 6-months data of the three groups on variables related to hepatic steatosis and fibrosis and associated parameters separately in patients with Crohn's disease.

		Timepoint	Controls (n=14)	Infliximab (n=12)	Vedolizumab (n=7)	p-value (between groups)	p-value for trend
Demographics	Age (years)		41.7 ±15.9	39.4 ±17.3	48.9 ±18.5	0.498	N/A
	Disease duration (years)		9.1 ±9.6	7.4 ±2.1	7.5 ±2.8	0.287	
	Body mass index (kg/m²)	Baseline	24.9 ±2.9	22.5 ±4.0	27.6 ±5.1	0.855	0.417
		Month 6	24.9 ±2.6	23.5 ±5.0	28.1 ±3.7	0.972	
	p-value (within groups)		0.963	0.138	0.583		
	Waist circumference (cm)	Baseline	91.4 ±15.1	98.7 ±15.6[a]	95.9 ±95.9	0.015	0.382
		Month 6	93.8 ±17.2	99.3 ±13.1	94.9 ±19.2	0.082	
	p-value (within groups)		0.067	0.673	0.705		
Disease activity	SES-CD	Baseline	9.1 ±8.7	8.6 ±2.9	7.14 ±3.49	0.275	N/A
	CDAI	Baseline	165.4 ±62.9	80.4 ±60.1[a]	100.71 ±111.04	0.023	0.991
		Month 6	-19.8 ±107.3	99.3 ±69.4[a]	15.14 ±75.89	0.019	
	p-value (within groups)		0.041	0.028	0.028		
Steatosis measurements	Controlled attenuation parameter (dB/m)	Baseline	251.1 ±58.5	231.0 ±84.6	279.5 ±47.1	0.624	0.555
		Month 6	224.5 ±39.4	225.7 ±51.6	279.0 ±45.1	0.823	
	p-value (within groups)		0.096	0.317	0.453		

	Fatty liver index	Baseline	35.2 ±17.7	21.7 ±18.3	44.0 ±37.8	0.125	0.731
		Month 6	36.9 ±15.5	26.1 ±28.9	41.4 ±30.9	0.198	
	p-value (within groups)		0.249	0.646	0.933		
	Hepatic steatosis index	Baseline	35.4 ±4.8	30.9 ±6.5	36.4 ±7.3	0.909	0.543
		Month 6	35.3 ±3.3	32.1 ±5.7	38.3 ±6.5	0.772	
	p-value (within groups)		0.976	0.405	0.253		
Liver fibrosis measurements	Liver stiffness (kPa)	Baseline	4.36 ±1.3	4.88 ±1.1	5.11 ±1.4	0.428	0.448
		Month 6	3.96 ±1.2	5.04 ±2.5	4.28 ±1.6	0.458	
	p-value (within groups)		0.392	0.698	0.097		
	FIB-4	Baseline	0.7 ±0.4	0.7 ±0.6	1.1 ±0.8	0.416	0.516
		Month 6	0.8 ±0.4	0.8 ±0.6	0.9 ±0.5	0.907	
	p-value (within groups)		0.048	0.534	0.735		
	NFS	Baseline	-3.2 ±1.4	-3.7 ±1.8	-2.7 ±1.8	0.453	0.505
		Month 6	-3.1 ±1.0	-3.3 ±1.4	-2.1 ±1.9	0.272	
	p-value (within groups)		0.730	0.182	0.310		
Laboratory tests	White blood cells (M/μL)	Baseline	8.5 ±2.7	10.6 ±3.6[a]	7.70 ±2.17	0.009	0.346
		Month 6	6.8 ±2.2	8.6 ±2.5	7.83 ±1.64	0.138	
	p-value (within groups)		0.019	0.120	0.900		
	Hemoglobulin (g/dL)	Baseline	13.1 ±1.3	12.5 ±1.8	12.66 ±1.33	0.623	0.292
		Month 6	13.3 ±0.8	13.4 ±1.5	12.83 ±1.14	0.603	
	p-value (within groups)		0.285	0.109	0.671		
	Platelets (K/μL)	Baseline	261.1 ±87.3	308.1 ±89.3	317.7 ±57.7	0.160	0.471
		Month 6	249.4 ±64.9	274.6 ±84.3	270.0 ±50.8	0.637	
	p-value (within groups)		0.433	0.272	0.063		
		Baseline	1.8 ±4.3	0.9 ±0.8	1.2 ±1.9	0.778	0.709

C-reactive protein (mg/dL)	Month 6	0.4 ±0.8	0.5 ±0.6	0.4 ±0.4	0.355	
p-value (within groups)		0.146	0.154	0.416		
Glucose (mg/dL)	Baseline	86.4 ±11.3	83.3 ±25.6	105.6 ±22.7	0.047	0.370
	Month 6	84.6 ±8.4	89.3 ±20.1	101.7 ±38.01	0.549	
p-value (within groups)		0.572	0.195	0.671		
Albumin (g/dL)	Baseline	4.2 ±0.6	4.3 ±0.3	4.3 ±0.4	0.921	0.760
	Month 6	4.5 ±0.3	4.4 ±0.3	4.4 ±0.2	0.835	
p-value (within groups)		0.100	0.063	0.671		
Aspartate aminotransferase (U/L)	Baseline	23.8 ±29.1	27.9 ±19.8	36.36 ±19.1	0.076	0.430
	Month 6	25.8 ±19.9	26.5 ±14.2	19.6 ±7.2	0.445	
p-value (within groups)		0.096	0.814	0.063		
Alanine aminotransferase (U/L)	Baseline	25.5 ±25.2	23.1 ±14.2	28.9 ±17.7	0.417	0.564
	Month 6	28.9 ±19.9	26.5 ±17.5	19.8 ±7.4	0.491	
p-value (within groups)		0.363	0.541	0.091		
Gamma-glutamyl transpeptidase (U/L)	Baseline	28.6 ±25.8	16.0 ±9.7	28.9 ±32.5	0.505	0.395
	Month 6	21.9 ±12.0	21.0 ±26.2	18.1 ±15.9	0.183	
p-value (within groups)		0.583	0.646	0.917		
Triglycerides (mg/dL)	Baseline	3.1 ±2.4	4.5 ±6.4	6.5 ±10.5	0.929	0.603
	Month 6	2.7 ±1.4	4.5 ±5.3	6.1 ±4.4	0.210	
p-value (within groups)		0.442	0.499	0.715		
Insulin (U/mL)	Baseline	13.9 ±10.4	18.7 ±15.9	20.2 ±35.4	0.339	0.706
	Month 6	12.6 ±6.2	14.8 ±11.7	23.8 ±18.8	0.261	

	p-value (within groups)		0.485	0.674	0.500		
	HOMA-IR	Baseline	3.3 ±2.2	4.4 ±5.8	3.7 ±6.7	0.227	0.955
		Month 6	2.7 ±1.6	3.3 ±3.9	4.6 ±3.9	0.340	
	p-value (within groups)		0.126	0.460	0.236		
	Adiponectin (μg/ml)	Baseline	12.1 ±5.7	10.7 ±1.7	8.8 ±3.6	0.492	0.737
		Month 6	13.3 ±7.6	10.6 ±3.4	9.6 ±6.9	0.593	
	p-value (within groups)		0.397	0.937	1.000		
	TNF (pg/ml)	Baseline	97.5 ±137.2	86.1 ±168.4	224.3 ±240.7	0.465	0.582
		Month 6	159.8 ±213.9	89.5 ±168.7	286.1 ±271.1	0.312	
	p-value (within groups)		0.347	0.182	0.075		
Serological markers	Leptin (ng/ml)	Baseline	10.3 ±3.0	10.92 ±2.8	13.49 ±2.9	0.089	0.842
		Month 6	10.8 ±3.5	12.07 ±4.2	13.98 ±3.7	0.252	
	p-value (within groups)		0.433	0.158	0.499		
	PIIINP (μg/L)	Baseline	5.5 ±2.7	6.9 ±10.9	9.7 ±13.6	0.599	0.485
		Month 6	10.5 ±11.1	8.8 ±10.1	11.4 ±12.7	0.833	
	p-value (within groups)		0.041	0.019	0.176		
	TIMP-1 (ng/ml)	Baseline	66.6 ±28.9	84.4 ±31.8	70.1 ±30.0	0.320	0.435
		Month 6	55.9 ±23.3	76.6 ±21.8	83.8 ±17.8a	0.020	
	p-value (within groups)		0.409	0.463	0.217		

Data are presented as mean± standard deviation. a: Compared to controls, b: Compared to infliximab

CDAI, Crohn's disease activity index; FIB-4, Fibrosis-4 index; HOMA-IR, Homeostasis Model Assessment of Insulin Resistance; NFS, NAFLD fibrosis score; PIIINP, N-terminal propeptide of procollagen type III; TIMP-1, tissue inhibitors of metalloproteinase 1; TNF, tumor necrosis factor

Supplementary Table S6. Comparative baseline and 6-months data of the three groups on variables related to hepatic steatosis and fibrosis and associated parameters separately in patients with ulcerative colitis.

		Timepoint	Controls (n=12)	Infliximab (n=14)	Vedolizumab (n=7)	p-value (between groups)	p-value for trend
Demographics	Age (years)		29.0 ±11.7	36.9 ±15.8	54.0 ±18.3[a.b]	0.042	N/A
	Disease duration (years)		4.4 ±6.8	6.3 ±9.5	13.9 ±15.2	0.099	
	Body mass index (kg/m^2)	Baseline	24.9 ±5.5	24.1 ±5.6	25.3 ±3.4	0.855	0.249
		Month 6	24.9 ±5.2	25.3 ±6.3	25.6 ±2.6	0.972	
	p-value (within groups)		0.973	0.040	0.707		
	Waist circumference (cm)	Baseline	81.9 ±10.6	99.4 ±15.9[a]	93.9 ±17.1	0.015	0.103
		Month 6	86.4 ±13.6	99.7 ±15.4	94.3 ±14.3	0.082	
	p-value (within groups)		0.029	0.771	0.857		
Disease activity	Mayo score	Baseline	5.2 ±3.5	9.6 ±1.6[a]	6.9 ±3.1	0.004	0.871
		Month 6	1.8 ±1.5	5.6 ±2.9[a]	4.0 ±4.0	0.032	
	p-value (within groups)		0.008	0.042	0.027		
Steatosis measurements	Controlled attenuation parameter (dB/m)	Baseline	259.9 ±84.1	239.4 ±54.8	226.0 ±45.5	0.624	0.990
		Month 6	238.2 ±79.7	228.8 ±36.5	211.0 ±62.8	0.823	
	p-value (within groups)		0.355	0.726	0.541		
	Fatty liver index	Baseline	37.3 ±28.4	28.9 ±26.8	36.4 ±26.4	0.704	0.697
		Month 6	34.0 ±30.9	30.5 ±29.1	38.4 ±25.1	0.703	
	p-value (within groups)		0.456	0.726	0.735		

Liver fibrosis measurements	Hepatic steatosis index	Baseline	33.3 ±6.2	34.3 ±11.3	32.6 ±5.4	0.909	0.852
		Month 6	32.6 ±5.8	34.5 ±9.0	32.9 ±4.6	0.772	
	p-value (within groups)		0.488	0.870	0.878		
	Liver stiffness (kPa)	Baseline	4.4 ±1.4	5.2 ±1.5	4.6 ±1.5	0.428	0.737
		Month 6	3.9 ±1.4	4.7 ±0.9	4.3 ±1.3	0.458	
	p-value (within groups)		0.589	0.361	0.766		
	FIB-4	Baseline	0.4 ±0.3	0.4 ±0.2	1.2 ±0.6[a.b]	0.001	0.254
		Month 6	0.5 ±0.6	0.6 ±0.3	1.6 ±1.3[a.b]	0.018	
	p-value (within groups)		0.025	<0.001	0.249		
	NFS	Baseline	-4.5 ±1.2	-5.3 ±2.7	-2.9 ±1.4[b]	0.043	0.455
		Month 6	-3.8 ±0.9	-3.9 ±1.3	-1.5 ±1.9[a.b]	0.009	
	p-value (within groups)		0.003	0.016	0.063		
Laboratory tests	White blood cells (M/μL)	Baseline	8.7 ±2.2	11.8 ±3.9[a]	7.4 ±2.9[b]	0.009	0.127
		Month 6	7.9 ±1.9	8.8 ±3.4	6.3 ±1.5	0.138	
	p-value (within groups)		0.373	0.003	0.233		
	Hemoglobulin (g/dL)	Baseline	13.0 ±2.6	12.1 ±2.3	13.3 ±1.1	0.292	0.678
		Month 6	12.9 ±2.5	13.0 ±0.9	13.9 ±0.9	0.499	
	p-value (within groups)		0.929	0.139	0.735		
	Platelets (K/μL)	Baseline	317.2 ±60.6	447.1 ±201.16	311.6 ±79.9	0.021	0.088
		Month 6	287.8 ±61.5	333.7 ±96.7	260.7 ±84.9	0.145	
	p-value (within groups)		0.019	0.004	0.128		
	C-reactive protein (mg/dL)	Baseline	1.4 ±2.3	5.65 ±9.6	1.73 ±3.8	0.122	0.211
		Month 6	0.5 ±0.9	0.514 ±0.5	0.67 ±1.2	0.785	
	p-value (within groups)		0.099	0.002	0.276		
	Glucose (mg/dL)	Baseline	86.4 ±11.2	88.0 ±17.8	85.3 ±12.6	0.997	0.569
		Month 6	85.1 ±9.3	86.0 ±8.7	73.7 ±31.3	0.879	
	p-value (within groups)		0.695	0.701	0.866		
	Albumin (g/dL)	Baseline	4.5 ±0.4	3.8 ±0.7[a]	4.13 ±0.5	0.015	0.028
		Month 6	4.6 ±0.2	4.4 ±0.3	4.37 ±0.4	0.132	
	p-value (within groups)		0.397	0.003	0.046		

	Aspartate aminotransferase (U/L)	Baseline	19.3 ±9.4	13.4 ±5.4	31.8 ±16.9[a.b]	0.002	0.231
		Month 6	20.8 ±7.2	23.7 ±8.6	34.9 ±37.2	0.711	
	p-value (within groups)		0.271	0.002	0.866		
	Alanine aminotransferase (U/L)	Baseline	18.6 ±11.6	16.9 ±16.4	22.6 ±6.7	0.095	0.077
		Month 6	18.2 ±8.0	24.7 ±16.2	19.5 ±10.66	0.532	
	p-value (within groups)		0.688	0.06	0.237		
	Gamma-glutamyl transpeptidase (U/L)	Baseline	14.1 ±9.0	26.07 ±23.6	15.67 ±8.6	0.355	0.191
		Month 6	11.9 ±4.9	14.14 ±9.3	20.17 ±11.8	0.317	
	p-value (within groups)		0.539	0.017	1.00		
	Triglycerides (mg/dL)	Baseline	72.0 ±22.5	99.9 ±93.5	107.8 ±42.3	0.185	0.721
		Month 6	81.5 ±45.5	92.7 ±41.8	124.8 ±72.5	0.450	
	p-value (within groups)		0.695	0.510	0.612		
	Insulin (U/mL)	Baseline	16.7 ±7.8	17.7 ±19.2	8.4 ±7.4	0.056	0.355
		Month 6	12.4 ±7.9	9.9 ±6.0	9.8 ±3.4	0.685	
	p-value (within groups)		0.142	0.050	0.109		
	HOMA-IR	Baseline	3.6 ±1.9	4.2 ±5.6	1.9 ±1.9	0.105	0.406
		Month 6	2.7 ±1.8	2.0 ±1.1	2.2 ±0.9	0.719	
	p-value (within groups)		0.182	0.080	0.102		
	Adiponectin (µg/ml)	Baseline	11.4 ±7.7	10.7 ±3.6	14.1 ±5.1	0.303	0.638
		Month 6	13.9 ±9.6	11.8 ±5.0	13.8 ±4.4	0.700	
	p-value (within groups)		0.480	0.245	0.237		
	TNF (pg/ml)	Baseline	181.2 ±195.6	179.6 ±191.9	17.6 ±25.9	0.081	0.505
Serological markers		Month 6	162.9 ±213.9	199.7 ±203.6	90.9 ±129.4	0.416	
	p-value (within groups)		0.241	0.279	0.068		
	Leptin (ng/ml)	Baseline	11.1 ±4.2	11.4 ±3.9	12.1 ±4.6	0.951	0.589
		Month 6	11.3 ±3.9	10.1 ±3.5	11.4 ±4.8	0.610	
	p-value (within groups)		0.308	0.331	0.866		
	PIIINP (µg/L)	Baseline	6.4 ±2.9	4.9 ±2.4	4.3 ±1.9	0.173	0.715

	Month 6	8.9 ±3.9	7.2 ±4.9	8.2 ±5.2	0.403	
p-value (within groups)		0.117	0.064	0.063		
TIMP-1 (ng/ml)	Baseline	60.1 ±23.9	99.0 ±40.3	65.9 ±25.2	0.011	0.284
	Month 6	52.3 ±26.9	71.9 ±32.3	64.1 ±16.3	0.211	
p-value (within groups)		0.409	0.060	0.788		

Data are presented as mean± standard deviation. a: Compared to controls, b: Compared to infliximab

FIB-4, Fibrosis-4 index; HOMA-IR, Homeostasis Model Assessment of Insulin Resistance; NFS, NAFLD fibrosis score; PIIINP, N-terminal propeptide of procollagen type III; TIMP-1, tissue inhibitors of metalloproteinase 1; TNF, tumor necrosis factor
